# Neonatal Lactic Acidosis Associated With Antiretroviral Exposure: A Case Report

**DOI:** 10.7759/cureus.109292

**Published:** 2026-05-20

**Authors:** Joanna Mercado, Laiza Rivera, Aisha Droz López, Yolymar Poventud, Cesar Andino-Colón

**Affiliations:** 1 Emergency Medicine, University of Puerto Rico, Medical Sciences Campus, San Juan, PRI

**Keywords:** antiretroviral therapy, emergency medicine, lactic acidosis, mitochondrial toxicity, zidovudine

## Abstract

Antiretroviral therapy (ART) prophylaxis in infants born to mothers with human immunodeficiency virus (HIV) has significantly reduced vertical transmission rates; however, these medications may cause severe adverse effects, including lactic acidosis. We present the case of a seven-day-old boy born at 37 weeks to an HIV-positive mother who presented to the emergency department with persistent irritability. The patient was receiving HIV prophylaxis with lamivudine, zidovudine, and nevirapine. He demonstrated persistent tachycardia despite an otherwise reassuring physical examination. Laboratory evaluation revealed elevated serum lactate levels with worsening hyperlactatemia on serial measurements. In coordination with the Infectious Disease team, the ART regimen was modified to raltegravir and lamivudine, resulting in metabolic improvement. This case underscores the importance of recognizing ART-related toxicity in neonates with progressive hyperlactatemia despite initially subtle clinical manifestations, prompting early treatment readjustment and close monitoring.

## Introduction

Vertical, or mother-to-child, transmission is the most common route of human immunodeficiency virus (HIV) infection in children globally [1]. Without antiretroviral therapy (ART) and other preventive interventions, the risk of perinatal transmission ranges from approximately 15% to 30% [2]. Mortality among youth with perinatally acquired HIV is significantly elevated, with cohort studies demonstrating an approximately six- to 12-fold increased risk of death compared with matched individuals in the general population [3].

The implementation of ART has led to a dramatic reduction in vertical transmission, with rates now estimated at ≤1% in high-resource settings [2]. While these risk-based strategies have been highly effective in preventing perinatal transmission, they expose neonates to medications associated with significant adverse effects [4]. As a result, emergency physicians are increasingly likely to encounter HIV-exposed neonates receiving antiretroviral prophylaxis, a population with unique clinical considerations that often present with subtle and nonspecific signs [5].

We present the case of a neonate who developed severe lactic acidosis while receiving combination ART, emphasizing the importance of maintaining a high index of suspicion for ART-related toxicity and timely diagnostic evaluation in the emergency department (ED) setting.

This work was previously presented as a meeting abstract at the 46th Annual Research and Education Forum of the University of Puerto Rico, Medical Sciences Campus, on March 27, 2026.

## Case presentation

A seven-day-old boy born at 37 weeks of gestation via uncomplicated cesarean delivery was brought to the ED by his parent due to persistent, inconsolable crying that began approximately six hours prior to arrival, along with concern for clear right eye discharge and absence of bowel movements for the preceding two days. The patient was born to a mother with HIV infection diagnosed during pregnancy; however, her treatment adherence and intrapartum management were unknown. Since birth, the patient had been receiving combination ART prophylaxis consisting of oral zidovudine 11 mg every 12 hours, lamivudine 5 mg every 12 hours, and nevirapine 18 mg every 12 hours, with a planned six-week treatment course. According to the parent, the infant had been feeding appropriately, consuming approximately two ounces of formula every two hours with normal urine output. The parent denied fever, vomiting, diarrhea, respiratory distress, rash, or lethargy. There were no known sick contacts.

On arrival, vital signs included blood pressure 73/35 mmHg, heart rate of 204 beats per minute, respiratory rate of 45 breaths per minute, temperature of 36.7°C, and oxygen saturation of 99% on room air. The patient's weight measurement was 3.18 kg (19th percentile for his age). Physical examination was unremarkable.

Due to suspicion of ART-associated lactic acidosis in the setting of irritability and tachycardia, serum lactate was obtained as part of the initial laboratory evaluation and was found to be elevated at 4.5 mmol/L. The remaining laboratory results, including a complete metabolic panel, a complete blood count, and urinalysis, were reassuring (Tables 1-2).

**Table 1:** Initial laboratory results

Test	Value	Normal Range
Lactic acid	4.5 mmol/L	0.5-2.2 mmol/L
Lactate dehydrogenase	449 U/L	225-600 U/L
Alkaline phosphatase	198 U/L	150-420 U/L
Sodium	134 mmol/L	135-145 mmol/L
Potassium	4.6 mmol/L	3.5-6.0 mmol/L
Chloride	105 mmol/L	98-113 mmol/L
CO_2_	19 mmol/L	20-28 mmol/L
Anion gap	14.6 mmol/L	8-16 mmol/L
Serum osmolarity	253 mOsm/kg	275-295 mOsm/kg
Albumin	3.0 g/dL	2.8-4.4 g/dL
Blood urea nitrogen (BUN)	3 mg/dL	2-20 mg/dL
Creatinine	0.42 mg/dL	0.2-0.9 mg/dL
Bilirrubin	7.91 mg/dL	<12 mg/dL
Lipase	63 U/L	0-160 U/L
Calcium	9.5 mg/dL	8.5-10.5 mg/dL
Total bilirubin	7.91 mg/dL	1.0-12.0 mg/dL
Carbon dioxide	19 mmol/L	20-28 mmol/L
Glucose	56 mg/dL	45-90 mg/dL
White blood cell count	8.10 ×10^3^/µL	5.0-20.0 ×10^3^/µL
Red blood cell count	4.34 ×10^6^/µL	4.0-6.0 ×10^6^/µL
Hemoglobin	15.5 g/dL	14-22 g/dL
Hematocrit	45.2%	42-65%
Mean corpuscular volume	104.2 fL	95-120 fL
Platelets	296 ×10^3^/µL	150-450 ×10^3^/µL

**Table 2:** Urinalysis

Test	Value	Normal Range
Color	Yellow	Yellow-amber
Appearance/clarity	Clear	Clear
Blood	Negative	Negative
Glucose	Negative	Negative
Bilirrubin urine	Negative	Negative
Ketone	Negative	Negative
Specific gravity	1.003	1.005-1.030
pH	6.5	4.6-8.0
Protein	Negative	Negative
Urobilinogen	0.2 E.U./dL	0.2-1.0 E.U./dL
Nitrite	Negative	Negative
Leukocyte esterase	Negative	Negative
White blood cell count	0-2/hpf	0-5/hpf
Red blood cell count	0-2/hpf	0-5/hpf
Squamous epithelial cells	Occasional	None-few
Casthyal	0-2/lpf	0-8/lpf
Bacteria	Few	None-few

**Figure 1:**
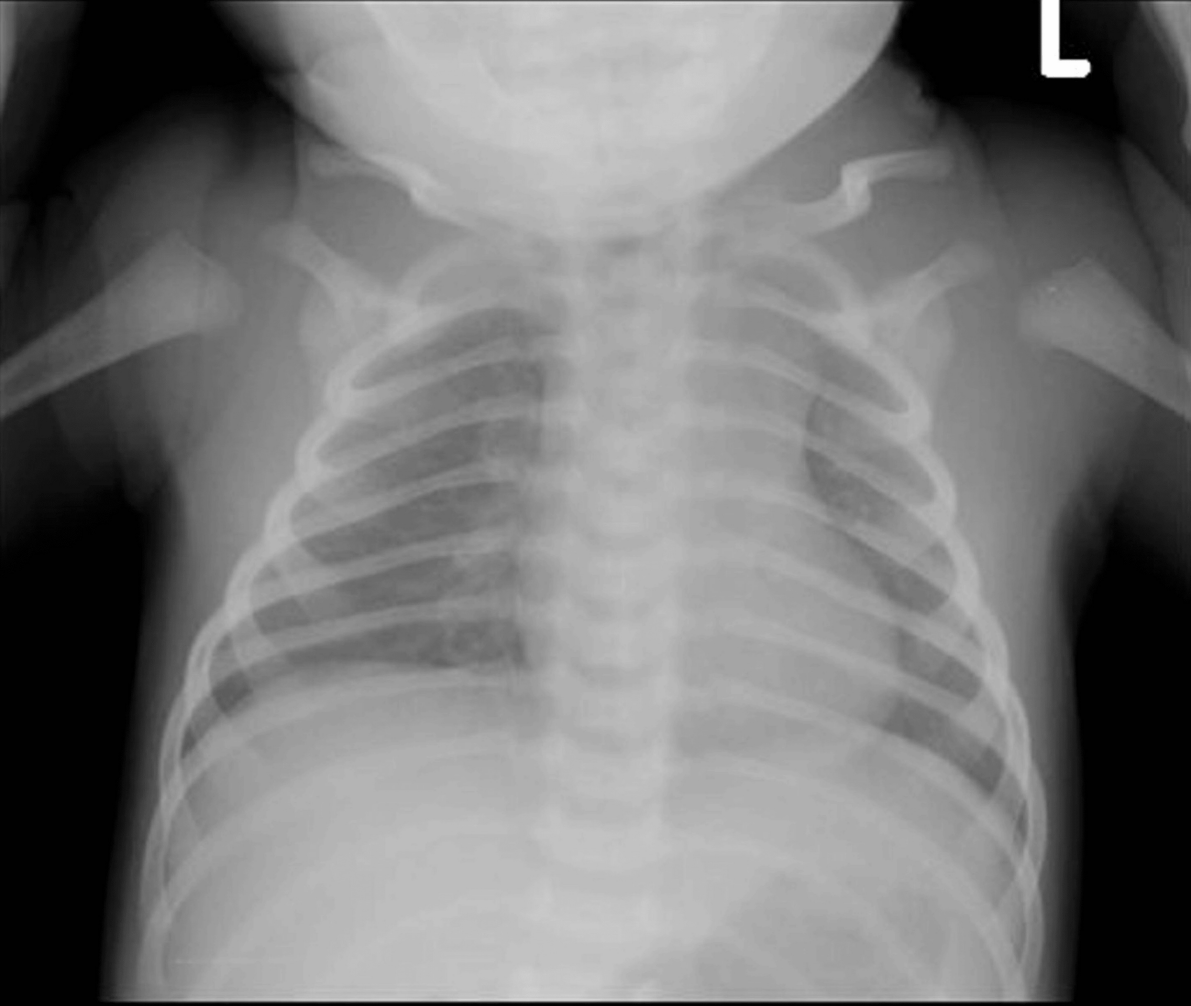
Neonatal chest radiograph Chest radiograph of a seven-day-old neonate demonstrating well-expanded lungs and no evidence of focal airspace opacities, pleural effusion, or pneumothorax.

**Figure 2:**
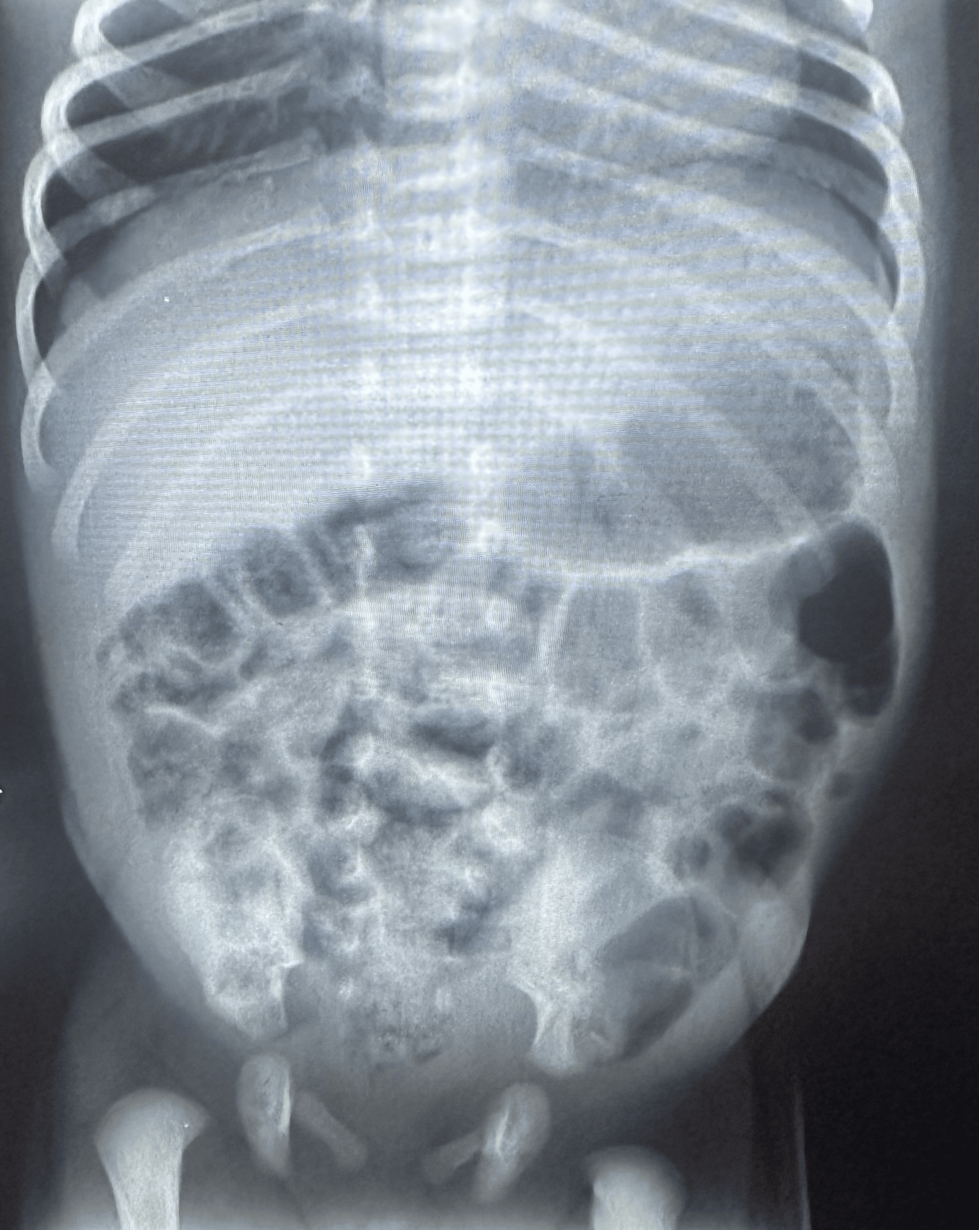
Neonatal abdominal radiograph Abdominal radiograph of a seven-day-old neonate without acute findings of obstruction, pneumatosis, or free intraperitoneal air.

Both chest and abdominal radiographs were unremarkable (Figures 1-2).

During his ED stay, the patient received supportive care, including intravenous fluids with interval improvement of heart rate to 122 beats per minute, blood pressure 72/51 mmHg, respiratory rate 26 breaths per minute, temperature 36.5°C, and oxygen saturation of 100%. Lactate levels six hours from arrival remained elevated at 4.1 mmol/L.

Given concern for evolving lactic acidosis, the patient was transferred to a tertiary pediatric facility for further evaluation and management. Upon arrival, 24 hours from the initial presentation, the physical examination was unremarkable. Repeat lactate levels at this time demonstrated an increase to 7.3 mmol/L in the absence of other notable laboratory derangements (Table 3).

**Table 3:** Follow-up laboratory results at 24 hours

Test	Value	Normal Range
Lactate	7.3 mmol/L	0.5-2.2 mmol/L
Sodium	138 mEq/L	135-145 mEq/L
Potassium	4.5 mEq/L	3.5-6.0 mEq/L
Chloride	106 mEq/L	98-110 mEq/L
Carbon dioxide	23.3 mmol/L	20-28 mmol/L
Blood urea nitrogen	3.5 mg/dL	2-20 mg/dL
Creatinine	0.442 mg/dL	0.3-1.0 mg/dL
Glucose	78 mg/dL	45-90 mg/dL
Calcium	9.2 mg/dL	8.5-10.5 mg/dL
Phosphorus	6.40 mg/dL	4.5-8.0 mg/dL
Ammonia	75.7 µmol/L	30-90 µmol/L
Procalcitonin	0.12 ng/mL	<0.5 ng/mL
C-reactive protein	0.4 mg/dL	<1.0 mg/dL

Following evaluation by the Infectious Disease service, zidovudine and nevirapine were discontinued, given suspected medication-induced mitochondrial toxicity contributing to lactic acidosis. The patient was transitioned to raltegravir while continuing lamivudine

At 48 hours from initial presentation, lactate levels further increased to 11.7 mmol/L. Venous blood gas analysis demonstrated low bicarbonate with hypocapnia and a near-normal pH, consistent with metabolic acidosis with respiratory compensation (Table 4).

**Table 4:** Venous blood gases (VBGs)

Test	Result	Normal Range
Venous blood gases pH	7.437	7.31-7.41
VBG pCO_2_	28.3 mmHg	40-50 mmHg
VBG HCO_3_	18.7 mmol/L	20-24 mmol/L
VBG O_2_ sat	99.3%	60-80%
Mixed VBG pO_2_	173.2 mmHg	30-50 mmHg

Blood cultures were negative. Last recorded vital signs included blood pressure 81/65 mmHg, heart rate 195 beats per minute, respiratory rate 39 breaths per minute, and temperature 36.9°C. The patient remained in the pediatric ward for close monitoring, during which serial lactate levels demonstrated gradual improvement to less than 3.5 mmol/L. The patient remained clinically stable and was discharged home within two days with outpatient follow-up in a specialized HIV clinic.

## Discussion

In neonates with elevated lactate levels, sepsis is a primary consideration due to impaired tissue perfusion and a shift toward anaerobic metabolism [6]. However, in this case, it was considered less likely given the absence of fever, hemodynamic instability, infectious focus, or elevated inflammatory markers [6]. Cardiac etiologies, such as congenital heart disease, were also less probable in the absence of respiratory distress, hepatomegaly, signs of poor perfusion, or abnormal cardiovascular findings [6,7]. Additionally, the normal neurologic examination made central causes of lactic acid elevation, such as seizures or acute neurologic injury, less likely [6]. The history of exposure to ART, progressive elevation in lactate levels, and relative hemodynamic stability supported ART-associated toxicity as the underlying etiology, favoring a type B lactic acidosis driven by impaired cellular metabolism rather than tissue hypoxia, characteristic of type A lactic acidosis [6,8].

A clear understanding of the pharmacologic mechanisms and toxicity profiles of neonatal ART regimens is essential for early recognition of therapy-related complications (Table 5). Nucleoside reverse transcriptase inhibitors (NRTIs) are synthetic deoxynucleoside analogues that require intracellular phosphorylation into active triphosphate forms, allowing competitive inhibition of viral reverse transcriptase [4,8]. However, these agents may also inhibit mitochondrial DNA polymerase-gamma, the enzyme responsible for mitochondrial DNA replication [4,8]. Resultant mitochondrial DNA depletion impairs oxidative phosphorylation and reduces respiratory chain protein synthesis, leading to decreased aerobic energy production and a compensatory shift toward anaerobic glycolysis with lactate accumulation [4,5,8]. This mechanism has been demonstrated in hepatic, cardiac, and skeletal muscle tissues [9]. The degree of toxicity varies among agents, with older NRTIs such as stavudine and didanosine associated with the highest risk, zidovudine demonstrating intermediate toxicity, and lamivudine and emtricitabine generally exhibiting lower mitochondrial effects [4,5,8]. Nevirapine is a non-nucleoside reverse transcriptase inhibitor (NNRTI) that exerts its antiviral effect through direct noncompetitive inhibition of HIV reverse transcriptase, resulting in conformational changes that impair viral DNA synthesis [4]. Unlike NRTIs, NNRTIs do not require intracellular phosphorylation and are not classically associated with direct mitochondrial polymerase-gamma inhibition [4,9]. Nevertheless, nevirapine has been linked to oxidative stress and hepatocellular injury through the formation of reactive metabolites capable of disrupting mitochondrial membrane integrity and promoting reactive oxygen species generation [10-12].

**Table 5:** ART prophylaxis regimens for neonates with in utero or intrapartum exposure to HIV: risk-stratified regimens, mechanisms of action, cellular mechanism of toxicity and adverse effects in neonates ART: antiretroviral therapy; NRTI: nucleoside reverse transcriptase inhibitor; NNRTI: non-nucleoside reverse transcriptase inhibitor; ATP: adenosine triphosphate

Risk Category	Recommended Regimen	Medication	Mechanism of Action	Cellular Mechanism of Toxicity	Adverse Effects in Neonates
Low risk	Monotherapy: 1 NRTI	Zidovudine (NRTI)	Zidovudine undergoes intracellular phosphorylation and inhibits HIV reverse transcriptase, resulting in premature viral RNA chain termination [13,14].	Inhibition of mitochondrial DNA polymerase-gamma leads to mitochondrial DNA depletion, impaired oxidative phosphorylation, reduced ATP production, and increased reactive oxygen species generation [15,16]. Accumulation of dysfunctional mitochondria further amplifies oxidative stress and promotes apoptotic cellular injury [17].	Anemia, neutropenia, thrombocytopenia, elevated liver enzymes, and lactic acidosis [13,18].
High risk	Combination: 2 NRTIs plus 1 NNRTI	Zidovudine (NRTI)	See above.	See above.	See above.
		Lamivudine (NRTI)	Lamivudine is a cytidine analog that inhibits reverse transcriptase via DNA chain termination [13,14].	Limited interaction with mitochondrial DNA polymerase-gamma [8,9]. Oxidative stress and mitochondrial dysfunction may still occur secondary to alterations in mitochondrial respiration [19].	Anemia, neutropenia, and lactic acidosis [18].
		Nevirapine (NNRTI)	Direct noncompetitive inhibition of HIV reverse transcriptase [14].	Hepatic bioactivation produces reactive metabolites that form protein adducts, leading to oxidative stress, mitochondrial injury, and immune-mediated apoptosis [10-12].	Transient asymptomatic lactatemia, anemia, neutropenia, hepatotoxicity, and hypersensitivity reactions, including rash [20].
Alternative (high risk)	Combination: 2 NRTIs plus 1 INSTI	Zidovudine plus Lamivudine	See above.	See above.	See above.
		Raltegravir (INSTI)	Raltegravir inhibits HIV integrase, preventing viral integration into human DNA [21].	Minimal mitochondrial toxicity at therapeutic exposure [22]. Limited interaction with uridine diphosphate-glucuronosyltransferase 1A1 (UGT1A1) may impair bilirubin conjugation, contributing to indirect hyperbilirubinemia, particularly in the setting of neonatal enzymatic immaturity [23].	Indirect hyperbilirubinemia and hepatic enzyme elevation [23].

ART-associated mitochondrial dysfunction presents along a spectrum ranging from asymptomatic hyperlactatemia to severe lactic acidosis [4,8,24]. Mild hyperlactatemia generally refers to elevated lactate levels without significant acid-base derangement, whereas lactic acidosis is typically characterized by lactate levels ≥5 mmol/L accompanied by metabolic acidosis [4,8]. This distinction is clinically relevant as isolated lactate elevation may be transient or clinically silent, while progressive lactate accumulation can reflect worsening mitochondrial dysfunction with the potential for rapid clinical deterioration [4,5].

In a cohort of 38 HIV-exposed but uninfected infants receiving perinatal ART, 92% of infants demonstrated at least one lactate measurement above 2.1 mmol/L, while 26% reached lactate levels ≥5 mmol/L [25]. All 38 infants tested negative for HIV polymerase chain reaction (PCR). Most infants remained asymptomatic despite lactate values as high as 7.4 mmol/L, and two neonates developed clinically symptomatic lactic acidemia at approximately two weeks of age, which presented with recurrent vomiting and irritability [25]. Both infants had been exposed to multidrug antiretroviral regimens in utero and received oral zidovudine during the neonatal period [25]. In response to concern for ART-related toxicity, zidovudine discontinuation resulted in symptom resolution and reduction of lactate levels to <4.2 mmol/L over the following two weeks [25]. Similarly, a 15-year cohort study of 96 ART-exposed neonates reported hyperlactatemia in 41.7% of patients, including 29.2% with isolated lactate elevation without acidosis, while 12.5% developed clinically significant lactic acidosis [26]. These findings suggest that mild lactate elevation is relatively common among ART-exposed neonates, whereas severe lactic acidosis is a less frequent manifestation [25,26].

Current guidelines stratify neonatal prophylaxis based on transmission risk [13]. Low-risk infants are defined as those born to mothers with sustained viral suppression, typically <50 copies/mL of HIV RNA from mid-pregnancy through delivery, with consistent prenatal care and adherence to ART [13]. These infants generally receive zidovudine monotherapy for two weeks, thereby limiting cumulative mitochondrial exposure [11]. In contrast, high-risk infants include those born to mothers with detectable viral load near delivery (≥50 copies/mL of HIV RNA), unknown maternal HIV status, inadequate prenatal care, or poor adherence to therapy [13]. These infants are usually treated with combination regimens including zidovudine, lamivudine, and either nevirapine or raltegravir for two to six weeks [13].

Although zidovudine remains a cornerstone of neonatal HIV prophylaxis, concerns regarding adverse metabolic effects and dosing complexity have led to increasing adoption of alternative regimens in high-risk neonates [4,5]. Among these, raltegravir-containing regimens have emerged as an attractive option since integrase inhibitors avoid direct inhibition of mitochondrial polymerase-gamma and demonstrate a more favorable safety profile compared with NRTI-predominant combinations [21]. Reported adverse effects of raltegravir are generally mild and primarily include indirect hyperbilirubinemia and reversible creatine kinase elevations [21-23].

In this case, incomplete maternal treatment history and unknown maternal viral load at delivery necessitated combination prophylaxis; however, cumulative exposure to multiple antiretroviral agents likely contributed to the development of hyperlactatemia and progressive metabolic dysfunction. In response to concern for ART-related toxicity, the regimen was transitioned to raltegravir and lamivudine, after which the infant demonstrated clinical stabilization.

Beyond currently recommended regimens, additional alternatives have also been explored in an effort to further improve safety and tolerability profiles in neonatal ART prophylaxis. Among these, tenofovir disoproxil fumarate (TDF)-based combinations have gained interest because of their comparatively lower mitochondrial toxicity relative to older NRTIs [27,28]. Available perinatal data remain generally reassuring, with first-trimester exposure to TDF and emtricitabine not associated with increased rates of congenital anomalies and overall acceptable renal, bone, and growth outcomes [27,29]. While some studies have reported small or transient effects on growth and bone parameters, current evidence suggests that TDF-based strategies may represent promising future alternatives in selected settings [27-29].

In this case, the rapid progression from mild hyperlactatemia in the ED to severe lactic acidosis emphasizes the importance of early lactate measurement and serial monitoring in neonates receiving ART. Rapid biochemical deterioration may occur despite relative clinical stability in ART-exposed neonates. Even modest lactate elevations should not be dismissed in this population, particularly when accompanied by otherwise unexplained clinical findings, as they may reflect evolving mitochondrial dysfunction and impending metabolic decompensation [24-26].

## Conclusions

In neonates receiving ART, nonspecific findings such as irritability or isolated tachycardia may be the only early indicators of evolving lactic acidosis. Even modest lactate elevations should prompt close reassessment and consideration of medication-related toxicity, as severe metabolic decompensation may develop rapidly. Therefore, continued evaluation of neonatal prophylactic regimens with improved safety and tolerability profiles is essential to reduce therapy-related complications.
